# From Inverse Problems in Mathematical Physiology to Quantitative Differential Diagnoses

**DOI:** 10.1371/journal.pcbi.0030204

**Published:** 2007-11-09

**Authors:** Sven Zenker, Jonathan Rubin, Gilles Clermont

**Affiliations:** 1 Center for Inflammation and Regenerative Modeling, University of Pittsburgh School of Medicine, Pittsburgh, Pennsylvania, United States of America; 2 Clinical Research, Investigation, and Systems Modeling of Acute Illness Laboratory, Department of Critical Care Medicine, University of Pittsburgh School of Medicine, Pittsburgh, Pennsylvania, United States of America; 3 Department of Mathematics, University of Pittsburgh, Pittsburgh, Pennsylvania, United States of America; Lilly Singapore Centre for Drug Discovery, Singapore

## Abstract

The improved capacity to acquire quantitative data in a clinical setting has generally failed to improve outcomes in acutely ill patients, suggesting a need for advances in computer-supported data interpretation and decision making. In particular, the application of mathematical models of experimentally elucidated physiological mechanisms could augment the interpretation of quantitative, patient-specific information and help to better target therapy. Yet, such models are typically complex and nonlinear, a reality that often precludes the identification of unique parameters and states of the model that best represent available data. Hypothesizing that this non-uniqueness can convey useful information, we implemented a simplified simulation of a common differential diagnostic process (hypotension in an acute care setting), using a combination of a mathematical model of the cardiovascular system, a stochastic measurement model, and Bayesian inference techniques to quantify parameter and state uncertainty. The output of this procedure is a probability density function on the space of model parameters and initial conditions for a particular patient, based on prior population information together with patient-specific clinical observations. We show that multimodal posterior probability density functions arise naturally, even when unimodal and uninformative priors are used. The peaks of these densities correspond to clinically relevant differential diagnoses and can, in the simplified simulation setting, be constrained to a single diagnosis by assimilating additional observations from dynamical interventions (e.g., fluid challenge). We conclude that the ill-posedness of the inverse problem in quantitative physiology is not merely a technical obstacle, but rather reflects clinical reality and, when addressed adequately in the solution process, provides a novel link between mathematically described physiological knowledge and the clinical concept of differential diagnoses. We outline possible steps toward translating this computational approach to the bedside, to supplement today's evidence-based medicine with a quantitatively founded model-based medicine that integrates mechanistic knowledge with patient-specific information.

## Author Summary

Although quantitative physiology has developed numerous mathematical descriptions of components of the human body, their application in clinical medicine has been limited to a few mostly primitive and physiologically inaccurate models. One reason for this is that the inverse problem of identifying unknown model parameters and states from prior knowledge and clinical observations does not usually have a unique solution. Hypothesizing that this non-uniqueness might actually convey clinically useful information, we used a simplified mathematical model of the cardiovascular system and its control, in combination with Bayesian inference techniques, to simulate the diagnosis of low blood pressure in an acute care setting. The inference procedure yielded a distribution of physiologically interpretable model parameters and states that exhibited multiple peaks. The key observation was that these peaks corresponded directly to clinically relevant differential diagnoses, enabling a quantitative, probabilistic assessment of the simulated patient's condition. We conclude that the proposed probabilistic approach to the inverse problem in quantitative physiology may not only be useful for quantitative interpretation of clinical data, and eventually allow model-based prediction and therapy optimization, but also provides a novel link between mathematically described physiological mechanisms and the clinical concept of differential diagnoses based on patient-specific information.

## Introduction

The amount of quantitative data available to the clinician at the bedside has grown tremendously because of advances in medical monitoring and imaging technology. This situation is particularly evident in the critical care setting, where patients are monitored and treatments are titrated on a minute-to-minute basis. The limit of currently available methods to assimilate this flood of data into the diagnostic and therapeutic process seems to have been reached, as suggested by the fact that an improved capacity to acquire quantitative measurements highly relevant for therapeutic decision making has failed to improve outcome [1,2]. The difficulty in translating richer data streams into improved clinical outcomes may be partly due to insufficient therapeutic options. We contend, however, that this failure may also be ascribed to human care providers' limited ability to integrate the sheer volume of available data and to quantitatively interpret the complicated and often nonlinear interactions of the various physiologic subsystems that contribute to these observations [3]. Additionally, the current evidence-based medicine paradigm, through its focus on validating therapeutic strategies for statistically identifiable subgroups of the population, limits the potential to provide truly individualized, validated care in data-rich environments like the intensive care unit [4–6]. This incongruence has clearly hampered more rapid progress in the care of the acute and critically ill, emphasizing the need for computerized data interpretation and decision support tools that make optimal use of the available information while enabling evidence-based validation of the decision making algorithm.

### Traditional Approaches to Computer-Supported Decision Making in Medicine

The potential of computer-based, algorithmic support for medical decision making in data-rich environments, and in particular in the context of evidence-based practice, was recognized early on and has been pursued extensively [3,7–11]. Few of these efforts, which mostly have consisted of rule-based expert systems, statistical models, or approaches driven by machine learning ideas such as dynamic Bayesian or artificial neural networks, have reached a sufficient level of practicality and usefulness to be accepted into the day-to-day practice of acute care medicine [8,12,13]. These tools either attempt to formalize empirical knowledge already available to a physician (expert systems) or to capitalize on statistical associations of phenomena and inherent structures of the available dataset. All largely fail to make direct and quantitative use of known causalities and dynamics in the physiologic systems underlying the observed pathophysiology, which are typically characterized by basic science investigations.

A promising approach to incorporating this knowledge into the medical decision making process would be to use mathematical models of physiologic mechanisms to map clinical observations to quantitative hypotheses about physiologic conditions, leading to improved insight into current patient status and, eventually, predictions about responses to therapeutic interventions. While complex mathematical models of physiology in general, and the cardiovascular system and its control in particular, have a long history and are still actively being developed [14–23], their translation to clinically useful tools has proved challenging. Early examples of using mathematical models to quantify “hidden” parameters based on clinical measurements include the pioneering work of Bergman et al. in the late 1970s on glucose control and insulin sensitivity [24]. More recent work in the same field has focused on accurately quantifying the uncertainty arising in the resulting parameter estimation problems using current methodology, such as Markov chain Monte Carlo approaches [25].

In the critical care environment, extremely simple models of the cardiovascular system have been in use for decades and are implemented in commercially available products, examples being the electric circuit analog of the systemic circulation used to calculate total peripheral resistance, which can then become a therapeutic target, or pulse contour analysis, which attempts a model-based assessment of systemic flow from arterial pressure waveforms [26–28]. The clinical application of mathematical models of physiology to date has failed to extend to models of sufficient complexity to significantly help alleviate the previously discussed problem of information overload in the diagnostic process.

### The Inverse Problem as an Obstacle to Application of Realistic Mathematical Models of Physiology

**Figure 1:**
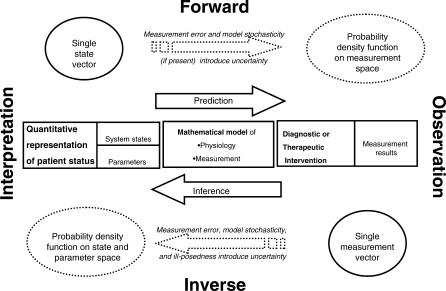
The Forward and Inverse Problems Illustration of the role of a mechanistic mathematical model in linking measurements with abstract quantitative representations of the underlying physiological processes. Sources of stochasticity are indicated both for forward/predictive use of the model and inverse/inference use. The forward problem consists of predicting (distributions of) observations if (distributions of) initial conditions and model parameters are known. The inverse problem refers to the task of inferring (distributions of) initial conditions and model parameters from observations. It is termed ill-posed if the available observations are insufficient to define a unique solution.

We contend that a key obstacle preventing the successful clinical use of available mathematical models has been the lack of a robust solution to the inverse problem. That is, any physiologically reasonable mathematical model of components of the human body will typically be nonlinear and have a large number of parameters. Despite the complexity of such models, if the user fixes the parameter values and initial values of the physiological states in a model, then the model can be simulated to obtain time courses of the physiological states (solving the forward problem; Figure 1). However, the corresponding inverse problem, i.e., inferring parameters and starting conditions of state variables from measured physiological data (http://www.ipgp.jussieu.fr/~tarantola/Files/Professional/Books/InverseProblemTheory.pdf ) [29] will usually be ill-posed in the sense of Hadamard, meaning that it does not admit a unique solution that depends continuously on the data [30–32] (Figure 1). This ill-posedness is directly related to the concept of system identifiability in both the statistical and engineering senses of the term. The most popular approaches to the inverse problem in physiology, such as nonlinear least squares, which seeks a maximum likelihood estimate by minimizing the sum of squared residuals, inherently assume the existence of a unique “best” solution. The ill-posedness of the inverse problem corresponds to a violation of this assumption, which often causes solution approaches such as least squares to fail completely, in spite of regularization of the underlying nonlinear programming problem, or to give meaningless or even misleading results. More recent work attempts to quantify the uncertainty of resulting parameter estimates [25]. However, given the uncertainty stemming from the fundamental ill-posedness of the inverse problem, together with additional uncertainty from measurement error and model stochasticity, which affect both forward and inverse problems (Figure 1), a fully probabilistic approach to the inverse problem in quantitative physiology seems appropriate.

### Ill-Posedness and Clinical Uncertainty

We hypothesize that the ill-posedness of the inverse problem is not merely a technical obstacle but reflects clinical reality in the sense that an experienced physician is rarely certain about a patient's status, despite a large number of available observations. More typically, the physician entertains an evolving differential diagnosis, consisting of a list of hypotheses of varying likelihoods about the physiological mechanisms underlying available observations, updated and ranked according to current observations. We therefore propose to approach the inverse problem in such a way that uncertainty from all sources is quantitatively reflected by the solution, which will consequently take the form of a (typically multimodal) probability distribution on parameter and state space. This distribution will represent the relative likelihoods of the possible values of the physiological elements that these parameters and states represent, in the patient for whom the clinical observations are made.

To explore the feasibility of such an approach, we combine a mechanistic model of cardiovascular physiology with a stochastic model of the observation process and Bayesian inference techniques to infer a posterior probability distribution on parameter and state space from prior (population-level and individual) knowledge and quantitative observations. We illustrate these ideas in a simplified simulation of a clinically relevant differential diagnostic procedure and examine the relationship between the obtained posterior probability density functions and pertinent qualitative differential diagnostic concepts.

## Results

**Figure 2:**
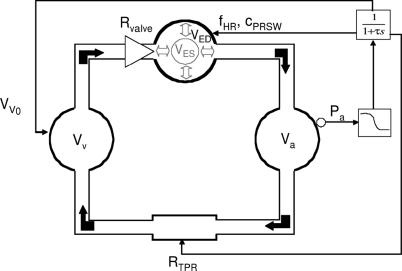
Schematic of the Simplified Model of the Cardiovascular System and Its Control Blood is driven from the venous compartment with volume *V*_v_ to the arterial compartment with volume *V*_a_ by the monoventricular heart, which contracts from its end-diastolic volume *V*_ED_ to its end-systolic volume *V*_ES_. Reverse flow is prevented by a valve with resistance *R*_valve_. To complete the systemic circulation, flow from the arterial to the venous compartment has to overcome the total peripheral resistance *R*_TPR_. Baroreflex senses pressure *P*_a_ in the arterial compartment, and it processes the set point deviation through a sigmoidal nonlinearity and a linear element with low-pass characteristics, eventually affecting the actuators *R*_TPR_, unstressed venous volume 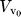 , heart rate *f*_HR_, and myocardial contractility *c*_PRSW_. See text for details.

**Figure 3:**
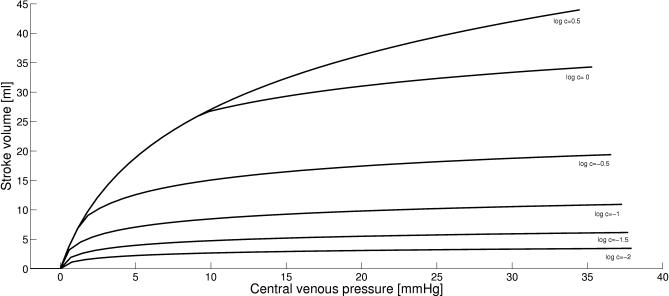
Starling Curves Steady state stroke volume with inactive baroreflex feedback loop as a function of venous pressure for various contractility factors *c* that linearly scale the range of the baroreflex contractility effector branch 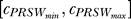 . Simulations were performed by varying total intravascular volume between 3,000 and 8,000 ml and plotting stroke volumes versus venous pressures after 600 s of simulated time.

**Figure 4:**
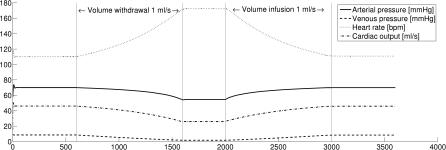
Simulation of Fluid Withdrawal and Reinfusion Fluid is drawn from or reinfused into the venous compartment at constant rate. Vertical lines indicate the beginning and end of withdrawal and reinfusion.

A simplified ordinary differential equation (ODE) model of the cardiovascular system was developed, including baroreflex blood pressure control, with specific focus on a correct representation of the interaction between myocardial contractility, intravascular volume, and peripheral resistance. We implemented an innovative conversion of the inherently discrete beat-to-beat cardiac dynamics to a continuous form, to allow for coupling to a continuous time representation of the relevant physiological control loops in a computationally efficient way. Simulation experiments reveal that the model, schematically depicted in Figure 2, exhibits qualitatively correct behavior with regard to the targeted clinically relevant physiological responses. Specifically, its steady state responses to alterations in both fluid load and contractility (Figure 3) agree with physiological expectations, and its blood pressure and heart rate responses, on medium-to-slow timescales, agree with the expected clinical response to dynamic challenges such as simulated volume loss (e.g., hemorrhage) and administration (e.g., fluid resuscitation; Figure 4).

A stochastic model of the observation process, simulating a symmetric Gaussian scatter with standard deviation σ of observed values around the true value, was used to represent imprecise measurements. Bayesian inference was used to sequentially assimilate observations on a specific hypothetical patient into probability density functions on a subset of lumped model parameters and states, which were assumed to vary in the patient population. We thus obtained a probabilistic estimate of the observed patient's condition in terms of the abstract physiological mechanisms represented by the model parameters and states.

The clinical scenario simulated and presented here consists of an initial observation of low blood pressure in the test patient, followed by a typical dynamical intervention, namely the intravenous application of fluid (fluid challenge; [33]). The virtual patient population was initially assumed to vary randomly with known distributions in two lumped parameters: hydration status (total intravascular volume) and myocardial contractility (scaling of contractility response range, two-dimensional setting; see Methods for details). We additionally explored the effects of allowing variation in peripheral vascular tone (scaling of total peripheral resistance response range, three-dimensional setting).

### Two-Dimensional Setting, Single Observation

**Figure 5:**
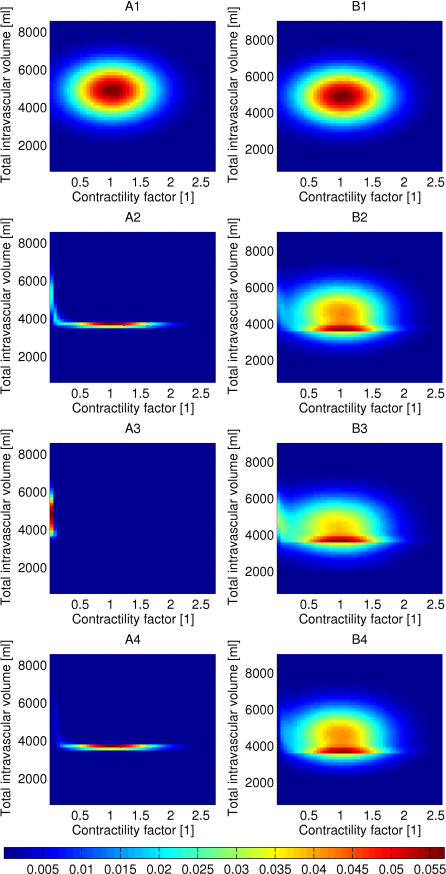
The Diagnostic Inference Process, Informative Priors Probability densities for high-precision (standard deviation 10 mm Hg) (A) and low-precision (standard deviation 30 mm Hg) (B) measurements of blood pressure for Gaussian prior densities. (A,B1) show the assumed prior densities, (A,B2) show the posterior densities resulting from a single arterial pressure measurement of 25 mm Hg, (A,B3) show the posterior densities if subsequent to the initial measurement, 1,500 ml of fluid are applied intravenously resulting in a pressure measurement of 30 mm Hg, while (A,B4) show the posterior densities if the measurement after fluid application is 70 mm Hg.

**Figure 6:**
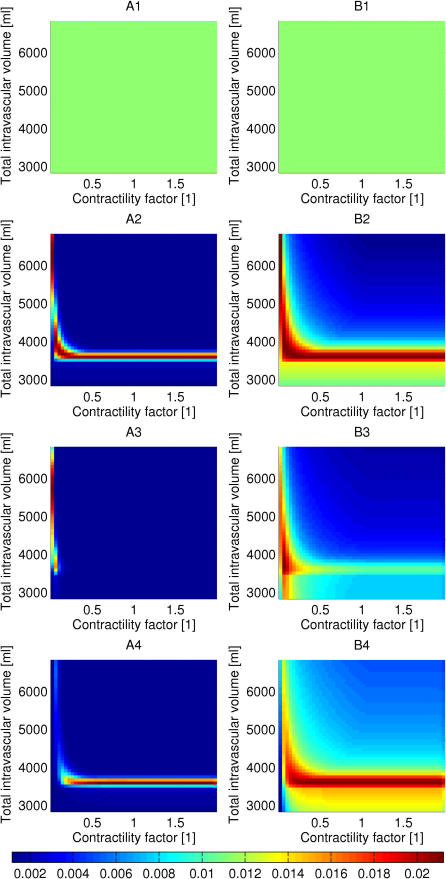
The Diagnostic Inference Process, Uniform Priors Probability densities for high-precision (standard deviation 10 mm Hg) (A) and low-precision (standard deviation 30 mm Hg) (B) measurements of blood pressure for uniform prior densities. The panel assignments are analogous to Figure 6. Note that the axes are scaled differently due to the narrower support of the (compactly supported) priors used.

When only one blood pressure measurement was made, the probable parameter/state range represented a continuum of various combinations of contractility and hydration status. As expected, high-precision measurements (σ = 10 mm Hg; Figures 5A and 6A) led to more concentrated probability density functions than low-precision measurements (σ = 30 mm Hg; Figures 5B and 6B), independent of the type of prior used. Two peaks, corresponding to the differential diagnoses of “heart failure” (low contractility, normal-to-high total intravascular volume) and “hypovolemia” (normal contractility, low intravascular volume), can be discerned in the case when blood pressure was measured with high precision for both Gaussian and uniform priors (Figures 5A2 and 6A2). When the measurement was less precise, the peak corresponding to “heart failure” was nearly absent with a Gaussian prior, but not with a uniform prior (Figures 5B2 and 6B2).

### Two-Dimensional Setting, Fluid Challenge

To illustrate the additional diagnostic knowledge gained from perturbing the system, we simulated a fluid challenge [33]. Depending on the system's response to the intravenous administration of 1,500 ml of fluid, the updated posterior densities on parameter space were altered significantly (Figures 5A3, 5A4, 5B3, 5B4, 6A3, 6A4, 6B3, and 6B4). Specifically, for a high-precision measurement, a fluid challenge differentiated between cardiac causes of hypotension (“heart failure”; low contractility, low responsiveness to volume resuscitation; Figures 5A3 and 6A3) and lack of intravascular volume as cause (“hypovolemia”; normal or high contractility, high responsiveness to volume resuscitation; Figures 5A4 and 6A4). With low-precision measurements, the failure to restore blood pressure following the fluid challenge did not eliminate hypovolemia as the cause of hypotension (Figures 5B3 and 6B3).

While the clinician often wonders whether there is a preferred sequence of diagnostic challenges for ascertaining an accurate diagnosis, the order of fluid challenges of different sizes, with consecutive assimilation of intermediate observations, had little effect on the final posteriors in this highly simplified setting.

### Three-Dimensional Setting, Fluid Challenge

**Figure 7:**
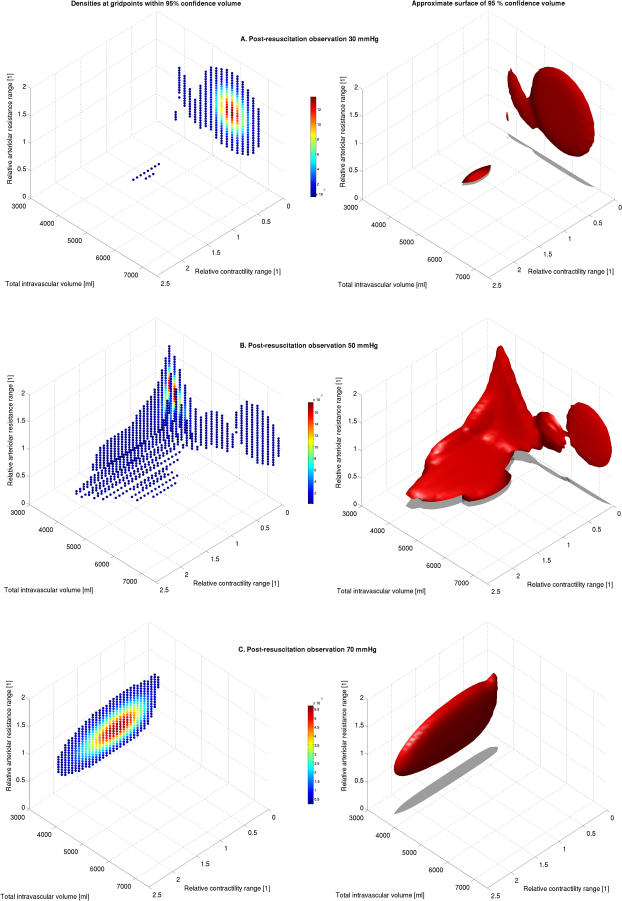
Three-Dimensional Inference Posterior probability densities for post-resuscitation observations of 30 mm Hg (A), 50 mm Hg (B), and 70 mm Hg (C) mean arterial blood pressure. The left column depicts densities at grid-points corresponding to 95% of the total probability mass, while the right column depicts the approximate surface enclosing this volume. The origin is in the far bottom corner for all figures. Shadows represent orthogonal projections to the contractility/total intravascular volume plane.

When we allowed three parameters to vary, the posterior distributions became truly multimodal. We depict two different visualizations of the grid points accounting for 95% of the total probability mass of posterior densities for the scenarios described earlier (Figure 7A and 7C), as well as for a more ambiguous post-resuscitation observation of 50 mm Hg (Figure 7B). As can be seen, the assimilated observations are still sufficient to meaningfully constrain the probable region in parameter/state space. In the poor (30 mm Hg post-resuscitation) response to volume scenario, an additional probability concentration appears. This additional probability mass corresponds to the possibility of shock induced by severely decreased peripheral resistance, which corresponds to the differential diagnostic possibility of a failure of vasomotor tone, as observed in septic, anaphylactic, or neurogenic shock states. For intermediate values of the post-resuscitation observation, the structure becomes even richer (Figure 7B), while the post-resuscitation observation of 70 mm Hg (good response) concentrates probability mass in a region of low intravascular volume (Figure 7C).

## Discussion

### Mathematical Model of the Cardiovascular System

While a greatly simplified physiological representation, our mathematical model of the cardiovascular system fulfills its design objectives: to be qualitatively correct in its response to variations in hydration status and myocardial contractility while incorporating enough homeostatic mechanisms to create realistic ambiguity in the identification of parameter values underlying observed states.

The conversion of the discrete dynamical system representing the sequential filling and emptying of the heart (and the resulting “history awareness” of the system) into a compact system of ODEs that preserves the physiologic meaning of parameters of the discrete system is, to our knowledge, novel. Physiologically constrained cardiovascular simulations done by previous authors have typically involved either simulating intra*-*beat dynamics, which rapidly becomes computationally prohibitive, using a more ad hoc approximation of the Starling mechanism at the expense of physiological interpretability of parameters, or resorting to a beat-to-beat discrete time representation (e.g., [20]). Our model is therefore particularly suited for simulation scenarios where an accurate description of intra*-*beat details is not required, yet a continuous form of inter*-*beat dynamics that preserves parameter meanings is desired.

Our model derivation aims to achieve a reasonable compromise between representing all known mechanisms in full physical detail, which leads to challenges of simulation expense and intractability of the inverse problem, and model reduction, which may result in loss of physiological accuracy and interpretability. The need for such a trade-off is typical when modeling complex biological systems. From our perspective, making use of domain-specific knowledge to arrive at meaningfully interpretable model reductions whenever possible, and resorting to multiscale models with a hierarchical arrangement of submodels of different granularity and timescales when the assimilation of data on very different spatial and temporal scales is desired, may be the most promising way to address this issue. The ideal level of model complexity will generally depend both on the amount of data available for assimilation and the specific application intended. To what extent the growing theoretical understanding of model selection based on information theoretical measures [34] can be leveraged to facilitate or partially automate this process for physiological applications is an interesting topic for further investigation.

### The Inference Process

As illustrated by this proof-of-concept implementation, the proposed methodology holds promise as a tool for integrating existing mechanistic knowledge and data generated by measurements in a clinical setting into a quantitative assessment of patient status. Our approach offers a means to achieve this integration in a way that not only incorporates all available data, but also quantifies the remaining uncertainty, thus avoiding unjustified claims of high certainty that could prove disastrous in a clinical setting. In particular, the clinical construct of differential diagnoses of different likelihoods is reflected in the observed multimodality of posterior probability distributions (Figures 5–7). More generally, representations of probability densities of states and parameters provide a natural setting for linking mathematical models of different scales and levels of detail since the distribution of states of some detailed small-scale models (“microstates”) may naturally determine a value or distribution of values for parameters of larger scale/lumped parameter models.

### Multimodal Posteriors and the Clinical Concept of Differential Diagnosis

The proposed approach aims to map clinical syndromes described by a set of observations to configurations of physiologically meaningful pre-observation states and parameters appearing within a mathematical model. Based on the physiological knowledge embodied in the model, certain regions in parameter and state space may in turn be associated with differential diagnoses, similar to the conditions of “hypovolemia,” “heart failure,” and “sepsis” in our simplified example. When this linkage is possible, the quantitative nature of the method presented here allows for the estimation and refinement of probability values associated with certain diagnoses. This, to our knowledge, is the first time that such a high-level concept central to clinical decision making is shown to emerge naturally from the combination of sequential observations, diagnostic challenges, and physiological principles. Moreover, we believe that the methods presented herein open novel avenues for exploring theoretical aspects of clinical epistemology, independent of practical applications.

Since measurement characteristics are described stochastically, the method we demonstrate is not fundamentally limited to assimilating data from device-based quantitative measurements, but can also make use of rather qualitative clinical observations such as quality of peripheral perfusion, presence of lung rales, or altered mental status, provided reasonably informative densities on system states or parameters conditional on such observations can be defined. Similarly, genomic information can be naturally incorporated, since it can provide probability distributions of physiological parameters conditional on individuals' genomes. To what extent a combination of several subjective (or inaccurate) observations may exploit physiological coupling of observables and yield informative posterior distributions corresponding, for example, to a carefully performed clinical examination is a matter of current investigation.

While modifying the order of physiological challenges did not have a tangible impact on diagnostic discrimination in our limited exploration, we anticipate that order generally matters, as a system's response to a perturbation can be highly dependent on the system state at the time that the perturbation is delivered. That is, an initial diagnostic challenge will alter the state of the underlying system, which may impact its response to a subsequent challenge. Our approach could allow for a theoretical exploration of how to optimize the selection, and order, of diagnostic challenges for maximal information gain in the context of specific clinical scenarios.

The simulations presented here illustrate the importance of congruence between the accuracy of observations and the level of information included in prior distributions. In particular, the inappropriate use of informative priors can be misleading in this context. In our results, for example, the combination of informative Gaussian priors with inaccurate observations effectively eliminates the physiologically reasonable “heart failure” peak in both the posteriors after a single observation of low blood pressure and the post-resuscitation posteriors for the low-response case, while the peak is still clearly evident in the case of uniform priors (Figures 5B2, 5B3, 6B2, and 6B3). This example demonstrates that a conscious choice needs to be made as to whether an interpretation based on population-level probabilities (corresponding to the use of informative priors) or an unbiased assessment of physiological possibilities (corresponding to the use of uniform priors) is more appropriate, when only few, low-quality measurements are available. Whether an optimal degree of incorporation of population-based information exists and how such an optimum could be defined are highly relevant issues that remain to be explored.

### Ill-Posedness of the Inverse Problem and Regulatory Mechanisms in Physiology

Our observations suggest that when addressing inverse problems in quantitative physiology, the traditional approach of requiring a unique optimal solution may be misleading and introduce unnecessary information loss, at least in situations where the additional computational burden of characterizing the posterior distributions more fully is not prohibitive. Model reduction to eliminate perceived “overparametrization” may weaken the correspondence of components of a physiologically faithful mathematical model with components of the actual physiological system it describes.

Furthermore, we hypothesize that the ambiguity of the mapping from observation to parameter space is at least partially due to a characteristic particular to physiological systems, namely their ability to tightly control certain system states via highly tuned, and often nested, internal regulatory feedback control mechanisms, such as the baroreflex in our simple example. In situations where such a controlled variable is observed, the ambiguity in the mapping from observation to parameter space is naturally exacerbated since perturbations of the observable will be compensated by alterations of other, possibly unobserved system states, as has already been proposed in a neurophysiological context [35]. Since a living organism is a system that maintains a state of dynamical equilibrium at energy expenditure, this phenomenon is likely to be the rule rather than the exception. A more precise formulation of this qualitative observation is the subject of current investigation.

### Future Challenges

#### Cardiovascular system model.

While the current system of ODEs appears suitable for the purposes of this report, further investigations relating the theoretical properties of the underlying hybrid system and of its continuous time approximation may yield deeper insights into the dynamics of the physiological system modeled and extend the applicability of the model. Furthermore, the relationship between the stability of fixed points of the isolated heart model and the full coupled system incorporating baroreflex warrants further investigation. Quantitative calibration and validation would naturally be desirable.

#### Clinical applicability of a probabilistic approach to inverse problems in mathematical physiology.

There are core theoretical and methodological challenges in expanding the proposed approach to realistic settings. A common aspect of most of these challenges is the “curse of dimensionality,” which is associated with the challenge of tackling high-dimensional problems in a computationally tractable fashion. Specific subproblems of immediate interest include the optimal inference of prior densities of parameters and system states from population-level data [36], the estimation of posterior densities from prior densities given current observations, the propagation of resulting state densities using estimated parameters, the visualization and computer-aided interpretation of high-dimensional posteriors, the extension of this methodology toward optimization of diagnosis and therapy, perhaps using control theoretical approaches, and, finally, the validation of the entire system or of some practical subcomponents, according to the criteria of evidence-based medicine. Recent methodological developments in the areas of sequential and Markov chain Monte Carlo methods [37,38] and sparse grids, and the continued exponential growth of available computational power, may contribute to making these steps feasible for reasonably sized models and datasets in the near future, possibly for real-time bedside use.

### Conclusion

We believe that our approach provides a conceptually new quantitative framework for a theoretical description of the development of differential diagnoses, which may potentially be harnessed to improve this process. Eventually, this methodology could be extended to an outcome prediction tool and could help to optimize diagnostic and therapeutic interventions in individual patients. Its practical implementation will require broad interdisciplinary collaborations, because of the significant challenges involved. We nevertheless believe that the potential gains in diagnostic effectiveness and efficiency that can be made by taking a quantitative approach to uncertainty, based on our ever-growing mechanistic understanding of physiology, will make the effort worthwhile.

## Methods

### A simplified mathematical model of the cardiovascular system and its regulation.

The model we developed was designed to be computationally and conceptually simple while achieving a good qualitative reproduction of system responses to alterations in contractility and hydration status. It consists of a continuous representation of the monoventricular heart as a pump, connected to a representation of the systemic circulation with the large blood vessels treated as linear capacitors (Windkessel model) and with arterial pressure controlled by a physiological feedback loop (baroreflex [39], Figure 2). The pulmonary circulation is excluded for simplicity, since the perturbations to be studied in our example are not directly related to it. The physiological variables and parameters used in the following exposition are summarized in Table 1.

**Table 1:**
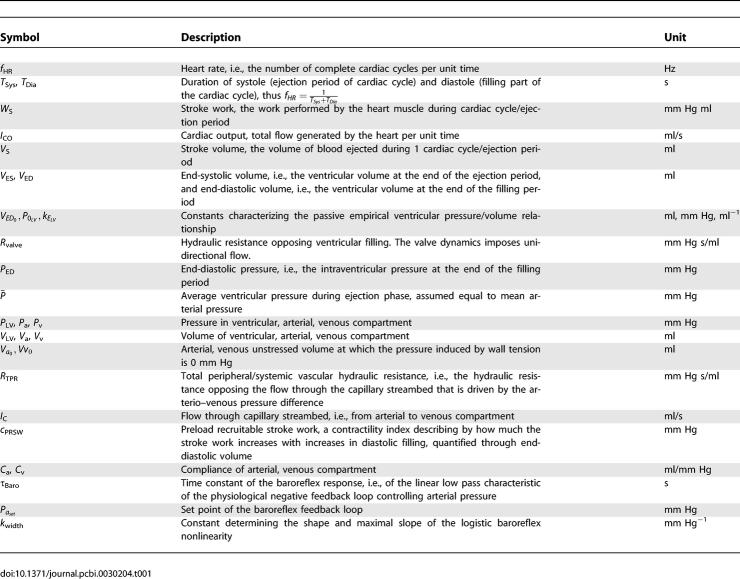
Glossary of Variables and Parameters of the Cardiovascular Model

***The heart as a pump.*** At a basic level, the heart acts analogously to a piston pump. During each heartbeat, blood from the venous side of the circulation fills the ventricle (“piston”) during the filling phase (diastole). When the cardiac rhythm generator (anatomically, the sinoatrial node) triggers myocardial contraction, the heart starts to contract, increasing the pressure in the ventricle. This process leads to the ejection of blood toward the arterial side of the circulation, and thus emptying of the ventricle, as soon as intraventricular pressure exceeds the pressure on the arterial side of the circulation. Simplifying the underlying physiology somewhat, one key factor for the amount of volume entering the ventricle in diastole is the so-called preload, which is related to the pressure in the large veins immediately upstream of the heart. How much blood is ejected during systole depends on the so-called afterload, corresponding to the pressure in the large arteries downstream of the heart, the strength of the contraction of the heart muscle (myocardial contractility), and the extent to which the ventricle was filled during diastole. The amount of force the myocardium can develop depends on its current level of stretch, which gives rise to a relationship between the amounts of filling during diastole and ejection during systole, termed the Starling mechanism. We developed an ODE model of the monoventricular heart, omitting pulmonary circulation, by considering a single-cycle representation of the emptying (ejection) and filling of the ventricle.

*Systole**.*** The model of ejection was based on the experimentally observed linearity of the relationship between stroke work *W*_S_, which refers to the work performed by the heart during ejection, and end-diastolic volume *V*_ED_ over a wide range of volumes [40]. This linear relation takes the form

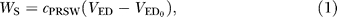
where the slope factor *c*_PRSW_ is termed the preload recruitable stroke work. The volume axis intercept of this relationship has been found to be equivalent to the volume at which the passive intraventricular pressure is 0 mm Hg, 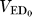 [40].

Approximating stroke work as pure volume work performed from *V*_ED_ to the end-systolic ventricular volume *V*_ES_ against the arterial pressure *P*_a_ yields

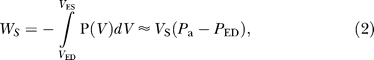
where *P*_ED_ is the intraventricular pressure at the end of diastole, and

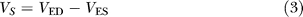
is the stroke volume. Based on the finding that the ventricular volume will not usually decrease below 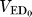 , we define the end-systolic volume as a function of the end-diastolic volume as follows:

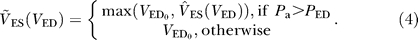
The expression for 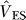 is obtained by equating the expressions for *W*_S_ in Equations 1 and 2, solving for *V*_S_, and substituting the result into Equation 3 to obtain

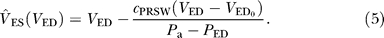
Note that 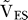 is a continuous function of *V*_ED_ since, if 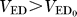 , the limit of 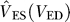 as *P*_ED_ approaches *P*_a_ from below is smaller than 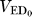 .

*Diastole*. To complete one stroke cycle, we derive an expression for the end-diastolic volume as a function of the end-systolic volume. Ventricular filling is modeled as a simple passive filling through the linear inflow resistance *R*_valve_, driven by the difference between pressure in the central veins *P*_CVP_ and ventricular pressure P_LV_(*V*_LV_), through the ODE

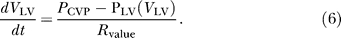
In Equation 6, the dependence of ventricular pressure on ventricular volume is governed by the experimentally characterized [40] exponential relationship:

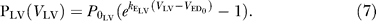



Under the assumption of constant *P*_CVP_, Equation 6 is of the general form

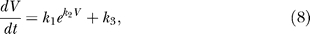
with constants

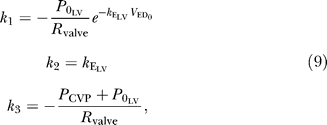
which resolves to

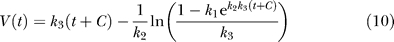
by quadrature. By letting *t* = 0 at the beginning of diastole and eliminating the unknown constant *C* using end-systolic volume *V*_ES_ as the initial condition, we obtain

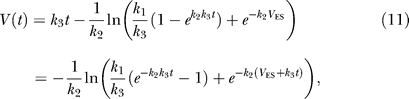
where the final expression is numerically advantageous since it avoids floating point overflow in the exponential terms. At a given heart rate *f*_HR_, and assuming an approximately constant duration of systole *T*_Sys_ (physiologically, the duration of diastole is much more strongly affected by alterations in heart rate than the duration of systole [41]), the end-diastolic volume will therefore be

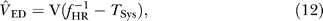
with V(*t*) given by Equation 11.

If *P*_CVP_ exceeds the intraventricular pressure at the beginning of diastole, then passive filling can occur and Equation 12 provides the desired expression for *V*_ED_ as a function of *V*_ES_, through the *V*_ES_-dependency of Equation 11. Otherwise, no filling will occur. The overall expression for *V*_ED_ as a function of *V*_ES_ is thus

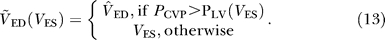
note that 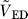 is a continuous function of *V*_ES_ since the limit of 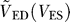 as P_LV_(*V*_ES_) approaches *P*_CVP_ from below is *V*_ES_.

*Joining systole and diastole**.*** We can now define a discrete dynamical system describing the beat-to-beat evolution of *V*_ED_ (or, similarly, *V*_ES_). Specifically, given the current end-diastolic volume 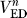 , we can use Equation 4 to compute 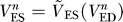 and use Equation 13 to obtain 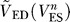 . Together, these steps yield

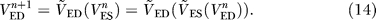



**Figure 8:**
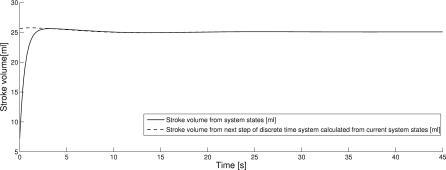
Transient Behavior of Continuous Time Cardiac Model Temporal evolution of actual stroke volume of continuous time system during initial transient of simulation shown in Figure 5 (solid line) and stroke volume calculated from systolic and end-diastolic volumes that would occur if the discrete dynamical system was advanced one step from the current values given by the continuous system (dashed line). Note that the state of the continuous system rapidly approaches a fixed point of the discrete dynamical system, resulting in superposition of the two curves. The transient is caused by starting integration with a non-equilibrium distribution of fluid between arterial and venous compartments.

To obtain a continuous dynamical system amenable to coupling with continuous representations of the physiologic control loops and simulation with available ODE software over long time intervals, we converted *V*_ES_ and *V*_ED_ to state variables of a continuous time system. This was done by setting their rates of change to the average rates of change over an entire cardiac cycle that would occur during one iteration of the discrete time system for the current *V*_ES_ and *V*_ED_ values, to obtain

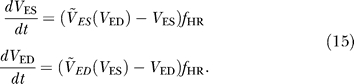
The discrete system (Equation 14) and the continuous system ( Equation 15) share identical sets of fixed points. Indeed, fixed points of the discrete system ( Equation 14) are given by

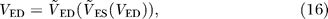
and by applying 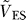 to Equation 16, we have

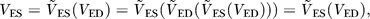
thus 
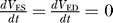
 at fixed points of the discrete system. Conversely, inspection shows that fixed points of Equation 15 satisfy Equation 16 and hence are fixed points of Equation 14 as well. The relationship between the stability of fixed points of Equation 14 and the stability of fixed points of Equation 15 is not obvious, since the discrete system (Equation 14) treats systole and diastole sequentially, while *V*_ES_ and *V*_ED_ co-evolve under Equation 15. However, linearization shows that any fixed point 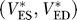 is stable with respect to both systems if 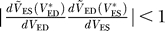 , whereas the fixed point is unstable with respect to both systems if 
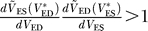
 . (The stability condition can be evaluated at all points in (*V*_ES_, *V*_ED_) space, except for on the finite collection of lines where either derivative fails to exist (see Equations 4 and 13). In the case that 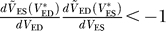 , the fixed point is stable with respect to the continuous time system and unstable with respect to the discrete, suggesting that the continuous approximation in theory has the potential to eliminate instabilities inherent in the beat-to-beat dynamics. However, preliminary numerical explorations suggest that 
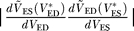
 is significantly smaller than one for the relevant parameter range. When the continuous system ( Equation 15) is embedded in the complete circulation model (described below), the full system very quickly settles to a stable fixed point (Figure 8).

*The systemic circulation.* The circulation is represented by a simple Windkessel model. It consists of linear compliances representing the large arterial vessels of volume *V*_a_ and venous vessels of volume *V*_v_ with respective pressures

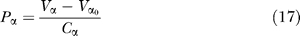
where α is “a” or “v” and where 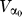 is the respective unstressed volumes, i.e., the in general non-zero volume at which the pressure in the respective compartment will be 0 mm Hg. These pressures appear in the equation that links the arterial and venous compartments through a linear resistor, representing the total peripheral resistance *R*_TPR_ regulating the arterio–venous capillary blood flow *I*_C_, namely

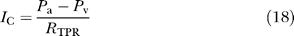



The veno–arterial flow (cardiac output) *I*_CO_ generated by the heart is given by the product of the heart rate *f*_HR_ and the volume *V*_S_ ejected per beat, which by Equation 3 takes the form

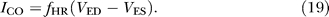



Assuming conservation of volume at the nodes, the evolution of arterial and venous volumes is described by the following differential equations:

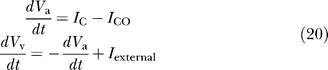
where *I*_external_ represents a possible external blood withdrawal or fluid infusion to or from the venous compartment.

*Baroreflex control of blood pressure.* Baroreflex control of blood pressure, which is one of the key regulatory mechanisms in cardiovascular homeostatis, is implemented based on the established representation of the central processing component of the baroreceptor sensor input as a combination of a sigmoidal nonlinearity (logistic function, in our case) with a linear system [15,18]. For simplicity, we reduced baroreflex activity to a single activating (sympathetic) output instead of the more physiologically accurate balance of stimulating (sympathetic) and inhibiting (parasympathetic) outputs. Since our model of the heart is designed to represent timescales significantly larger than a single beat, the linear part of the baroreflex feedback loop is simplified to display first-order low-pass characteristics with a time constant on the order of the slowest actuator response (unstressed venous volume control). Pure delays associated with the neural transmission of baroreflex signals are neglected. Under these assumptions, the temporal evolution of the stimulating output from baroreflex central processing is governed by the differential equation

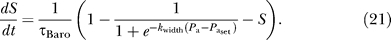



The stimulating output S(*t*) of the feedback loop acts on heart rate *f*_HR_, total peripheral resistance *R*_TPR_, myocardial contractility *c*_PRSW_, and unstressed venous volume 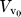 effectors/actuators to adjust blood pressure according to its current deviation from the set point, based on the linear transformations

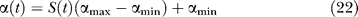
where α = *f*_HR_, *R*_TPR_, or *c*_PRSW_, and

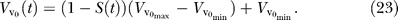
The form of Equation 23, in particular, arises since the venous capacitance vessels contract, reducing their unstressed volume, in response to drops in blood pressure.

Combining Equations 15 and 17– 23, and writing out dependencies relevant to the coupling of the system explicitly, we obtain a system of five ODEs:

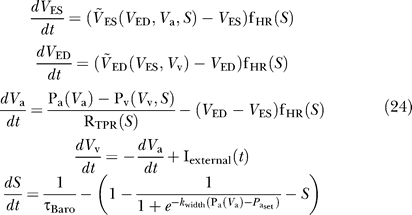
It should be noted that for *I*_external_ = 0, conservation of total intravascular volume would allow for elimination of one state variable (either *V*_a_ or *V*_v_) to obtain a four-dimensional system. We chose to leave the system in the above form, however, to preserve direct correspondence between anatomical entities and mathematical representation, at the cost of some loss in computational efficiency. With regard to the coupling between equations, it should be noted that the sympathetic nervous system activity *S*, which serves as a central control mechanism critical for functional cardiovascular system homeostasis, links together all components, while the coupling between the equations describing heart and circulation reflects the cyclical structure of the cardiac action and the circulation. Numerical solution of system Equation 24, as well as all other algorithms used for this work, was implemented in the MATLAB 7 (The MathWorks) programming environment, using the ode15s solver for numerical integration. The source code used in generating results is available in Text S1.

**Figure 9:**
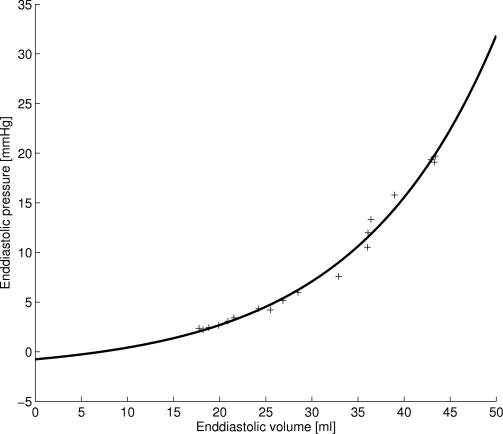
End-Diastolic Pressure–Volume Relationship Least squares fit of the empirical exponential pressure–volume relationship used to determine parameters from the experimental end-diastolic measurements from [40], Figure 6, bottom right panel.

*Parameter selection.* The parameters 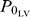 = 2.03 mm Hg, 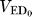 = 7.14 ml, and 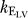 = 0.066 ml^−1^ describing the ventricular pressure–volume relationship were estimated from experimental data for the left ventricle from [40] using the Levenberg-Marquard nonlinear least squares algorithm (Figure 9).

The remaining parameter values and ranges for variables, as well as the respective sources, are given in Table 2 (for meaning of parameters, see Table 1). When no explicit source is given, assignment was based on the authors' perception of physiologically reasonable values for the simplified system, without claiming quantitative accuracy.

**Table 2:**
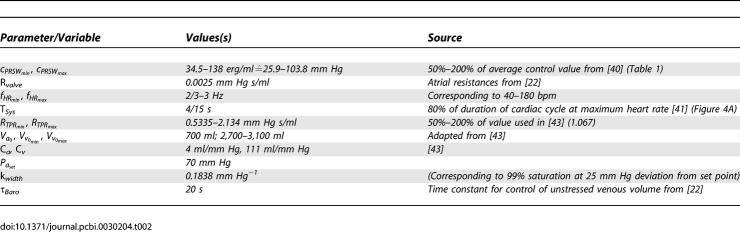
Parameter Values for the Model of the Cardiovascular System and their Sources

### The inference procedure.

For the purpose of this exposition, we are lumping the subset of *n* initial conditions (states) and parameters with which the inference is concerned into one product vector space with elements *x* ∈ *X* ℜ*^n^*. Additionally, for simplicity, we assume a fully deterministic model *M*: *X* →, *x* ↦; *M*(*x*) of the physiological process that gives a mapping from *X* to a finite dimensional observation space with elements *y* ∈ *Y* ℜ*^m^*. Extending the methodology to stochastic process models is straightforward but computationally more burdensome.

*Generation of prior densities.* In a practical application, prior probability densities on parameter/initial condition space would have to be inferred from a finite set of observations of a population, which in itself constitutes an ill-posed inverse problem. For simplicity, we assume a known prior distribution *f_X_* : *X* → [0,1] on parameter/initial condition space and compute an approximation to the prior density on observation space consistent with the mechanistic model by evaluating

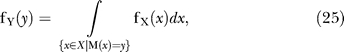
using a Monte Carlo approach. Specifically, we draw *N* = 10^2dim(*X*)^ samples *x_i_* from *f_X_* and compute the corresponding observations *y_i_* = *M*(*x_i_*). A histogram with 50 bins/dimension is computed for approximate evaluation of *f_Y_*.

In the two-dimensional simulations, the parameter/initial condition space *X* consisted of the total intravascular volume *V*_total_ = *V*_a_ + *V*_v_, corresponding to hydration status, and a positive scaling constant *c* applied to the cardiac contractility response range 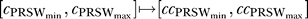 . Initial conditions for compartmental volumes were generated from *V*_total_ by setting them proportional to the unstressed volume of the respective compartment and ensuring that observations were taken only after the system had equilibrated. In the three-dimensional setting, an additional scaling factor 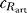 for the arteriolar resistance range 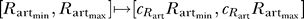 was introduced.

The prior densities *f_X_* for the informative/Gaussian case were independent Gaussian distributions with mean one and standard deviation 0.5 for the scaling constants, while the mean of the distribution of total intravascular volume was chosen to correspond to a level of sympathetic nervous activity S(*t*) ≈ 0.5 in steady state with both scaling factors at one, with a standard deviation of 1,000 ml. All distributions were truncated at zero by repeating the sampling if a negative value was drawn. *f_X_* for the uniform/non-informative case were independent uniform distributions on the intervals two standard deviations above and below the means of the corresponding Gaussian distributions, again truncated at zero.

*Sequential assimilation of observations.* Sequential updating of the posterior densities was accomplished based on the standard Bayesian calculation of the conditional density

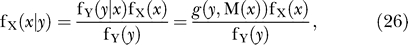
where the stochastic measurement model *g*: *Y* × *Y* → [0,1], (*y*,*y*_true_) ↦ *g*(*y*,*y*_true_) describes the distribution of observations as a function of the true value of the observable *y*_true_. In Equation 26, the prior distributions *f_X_* and *f_Y_* are either the original prior distributions described in the previous paragraph, if the first observation is being assimilated, or the posterior distribution on *X* obtained in the preceding assimilation step, approximated using weighted Gaussian kernel density estimation as implemented in the KDE toolbox for MATLAB [42], and its corresponding consistent distribution on *Y*, computed as described in the previous paragraph, in all other cases. For all of the above steps, visual comparisons between simulations with different and incommensurate grid resolutions were performed to ascertain that the observed multimodality of the distributions did not in fact constitute an artifact resulting from aliasing or other numerical effects.

## Supporting Information

Text S1 Code This file contains the MATLAB routines used to generate the results. Please see the README file in the archive for installation and usage instructions.
(21 KB GZ).

## Abbreviations

ODE: ordinary differential equation
