# Correction: From Inverse Problems in Mathematical Physiology to Quantitative Differential Diagnoses

**DOI:** 10.1371/journal.pcbi.1007155

**Published:** 2019-06-24

**Authors:** Sven Zenker, Jonathan Rubin, Gilles Clermont

In the Methods section, there is an error in the ninth numbered mathematical expression in the section titled “A simplified mathematical model of the cardiovascular system and its regulation.” The equation for *k*_*3*_ has an incorrect sign. Please view the complete, correct set of equations here:

$$k_{1}=-\frac{P_{0_{LV}}}{R_{valve}}e^{-k_{E_{LV}}V_{ED_{0}}}$$

$$k_{2}=k_{E_{LV}}$$

$$k_{3}=\frac{P_{0_{LV}}+P_{CVP}}{R_{valve}}$$

The correct expression was used in all derivations and simulations presented in the paper and thus this error does not, as far as the Authors are aware, affect any of the results or conclusions in the paper. The Authors regret any inconvenience this error may have caused.
